# Improved Immune Responses Against Zika Virus After Sequential Dengue and Zika Virus Infection in Humans

**DOI:** 10.3390/v10090480

**Published:** 2018-09-07

**Authors:** Félix G. Delgado, Karina I. Torres, Jaime E. Castellanos, Consuelo Romero-Sánchez, Etienne Simon-Lorière, Anavaj Sakuntabhai, Claude Roth

**Affiliations:** 1 Grupo de Virología, Universidad El Bosque, Bogotá D.C. 110131, Colombia; fdelgadot@unbosque.edu.co (F.G.D.); karinatorrescaballero@gmail.com (K.I.T.); castellanosjaime@unbosque.edu.co (J.E.C.); 2 Functional Genetics of Infectious Diseases Unit, Institut Pasteur, 75015 Paris, France; etienne.simon-loriere@pasteur.fr (E.S.-L.); anavaj.sakuntabhai@pasteur.fr (A.S.); 3 Unidad de Investigación Básica Oral, Universidad El Bosque, Bogotá D.C. 110131, Colombia; spacolombia@gmail.com; 4 CNRS UMR 2000: Génomique Évolutive, Modélisation et Santé, Institut Pasteur, 75015 Paris, France

**Keywords:** dengue virus, zika virus, T-cell epitopes, cross-reactive T cells, immunodominance, neutralizing antibodies, antibody-dependent-enhancement (ADE)

## Abstract

The high levels of dengue-virus (DENV) seroprevalence in areas where the Zika virus (ZIKV) is circulating and the cross-reactivity between these two viruses have raised concerns on the risk of increased ZIKV disease severity for patients with a history of previous DENV infections. To determine the role of DENV preimmunity in ZIKV infection, we analyzed the T- and B-cell responses against ZIKV in donors with or without previous DENV infection. Using peripheral blood mononuclear cells (PBMCs) from donors living in an endemic area in Colombia, we have identified, by interferon (IFN)-γ enzyme-linked immunospot (ELISPOT) assay, most of the immunodominant ZIKV T-cell epitopes in the nonstructural (NS) proteins NS1, NS3, and NS5. Analyses of the T- and B-cell responses in the same donors revealed a stronger T-cell response against peptides conserved between DENV and ZIKV, with a higher level of ZIKV-neutralizing antibodies in DENV-immune donors in comparison with DENV-naïve donors. Strikingly, the potential for antibody-mediated enhancement of ZIKV infection was reduced in donors with sequential DENV and ZIKV infection in comparison with donors with DENV infection only. Altogether, these data suggest that individuals with DENV immunity present improved immune responses against ZIKV.

## 1. Introduction

The Zika virus (ZIKV) is a flavivirus transmitted by *Aedes* species mosquitoes. It is a single positive-stranded RNA virus closely related to the yellow-fever virus, dengue virus (DENV), and West Nile virus [1]. Initially isolated in the Zika forest in Uganda in 1947 [2], it caused an explosive outbreak for the first time in Yap Island, Federated States of Micronesia in 2007 [3]. Subsequent outbreaks with higher number of cases occurred in 2013–2014 in French Polynesia and other South Pacific Islands, and, more recently, in the Americas [4,5,6,7,8,9]. Although initially believed to only cause mild, self-limiting disease, a causal relationship between ZIKV and neurological complications, such as Guillain-Barré syndrome or congenital malformations, was established during the 2013 and 2015 outbreaks in French Polynesia and Brazil [10,11,12,13].

While mutations in ZIKV genome might have contributed to its increased pathogenicity or explosive spread [9,14,15], one of the most important concerns today is related to the high level of DENV seroprevalence in areas where ZIKV is circulating [16]. Indeed, recent studies have shown that anti-DENV antibodies may enhance ZIKV infection and increase disease severity [17,18,19,20,21]. Given these constraints, and the lack of appropriate treatment for ZIKV infection, there is an urgent need to develop a vaccine against this infectious disease.

While antibodies against the E protein of DENV or ZIKV were shown to be highly cross-reactive, T cells can be cross-reactive or not, depending on the targeted peptides. A low degree of CD4 T-cell cross-reactivity between DENV and ZIKV was indeed observed in human donors immune to one of these viruses [18], whereas DENV/ZIKV cross-reactive T cells were identified in humans and in DENV-immune mice after challenge with ZIKV [22,23,24]. Considering the sequence identity between DENV and ZIKV for the structural proteins capsid and envelope, and the nonstructural proteins NS3 and NS5, which represent the main targets of DENV-specific CD4 and CD8 T cells, respectively, and the protective role of DENV-specific T cells [25,26], efforts are currently directed towards the mapping of T-cell epitopes to design new and more effective vaccines against ZIKV [27]. Predictions of T-cell antigens have been conducted by modeling potential epitopes from the ZIKV proteome that could bind to different HLA class I or class II alleles [23,28,29,30], or by analyzing ex vivo T-cell responses in transgenic mice expressing human HLA-B*07:02 and HLA-A*01:01 molecules [23]. More recently, ZIKV epitopes targeted by CD4 and CD8 T cells have also been identified from human donors living in ZIKV- and DENV-endemic regions [22]. Quite unexpectedly, while the majority of T-cell responses observed upon infection with DENV were directed against the nonstructural proteins NS3, NS4B, and NS5, ZIKV-specific T cells preferentially recognize structural proteins E, prM, and C, with conserved epitopes between DENV and ZIKV representing the main targets for cross-reactive T cells [22,23]. Furthermore, in the light of the recent identification of DENV/ZIKV cross-reactive T cells in human and in different animal models [22,24,31,32,33,34], the precise identification of ZIKV T-cell epitopes in the human that activate these cross-reactive T cells is essential to assess the role of these T cells in ZIKV infection and disease. In the present study, we have identified these epitopes from blood donors with a history of ZIKV-only infection or both DENV and ZIKV infection.

Using PBMCs from Colombian blood donors with previous ZIKV infection, we have first established a detailed map of the distribution of ZIKV T-cell epitopes, by quantifying ex vivo IFN-γ responses against peptides covering the whole ZIKV proteomic sequence by enzyme-linked immunosorbent spot (ELISPOT) assay. Measurement of the magnitude of T-cell responses (mediated by CD4 and/or CD8 T cells) against these peptides allowed us to identify immunodominant epitopes that induce strong responses in donors carrying specific HLA alleles. More specifically, we show that the nonstructural proteins NS1, NS3, and NS5 contain most of the immunodominant epitopes that induce a strong T-cell response. In donors with a history of DENV infection, the strongest T-cell responses were directed against peptides of the NS5 protein with a high level of amino acid identity with the four serotypes of DENV, and some matched previously described DENV CD8^+^ T-cell epitopes, suggesting the activation of cross-reactive T cells.

The neutralizing or enhancing activity of ZIKV-induced antibodies from the same donors were also analyzed using a flow cytometry-based assay. Results show that ZIKV infection in DENV-immune individuals resulted in increased levels of neutralizing antibodies against ZIKV, in comparison with DENV-naïve individuals, and in reduced potential for antibody enhancement of ZIKV infection, in comparison with DENV-immune ZIKV-naïve individuals. Altogether, these data strongly suggest that a sequential DENV and ZIKV infection may improve the immune protection against ZIKV infection but not against DENV infection.

## 2. Materials and Methods

### 2.1. Ethics Statement

Human blood samples were taken after obtaining the informed consent from healthy adult donors from the Fundación Hematológica Colombia (Bogotá D.C., Colombia) in accordance with the tenets of the Declaration of Helsinki. All protocols described in this study were approved by the institutional review board (IRB) of the EL Bosque University (Colombia). Donors were of both sexes and between 20 and 60 years of age. A total of 82 samples were obtained from different ZIKV-endemic areas near Bogotá D.C. (mainly from Villavicencio, Meta) between September and November 2016.

### 2.2. Human Blood Samples

PBMCs were purified by density gradient centrifugation (Lymphoprep™, Stemcell Technologies, Vancouver, BC, Canada), resuspended in fetal bovine serum (FBS) (Gibco Invitrogen, Carlsbad, CA, USA) containing 10% dimethyl sulfoxide and cryopreserved in liquid nitrogen. Eleven of the 82 blood samples obtained had to be excluded from the study due to poor viability of cells.

### 2.3. Viruses and Cell Lines

The in vitro assays were conducted using the DENV1 KDH0026A strain (provided by Dr L. Lambrechts, Institut Pasteur, Paris), DENV2 R0712259 strain (provided by Dr. A.-B. Failloux, Institut Pasteur, Paris), DENV3 KDH0010A strain (provided by Dr. L. Lambrechts, Institut Pasteur, Paris), DENV4 VIMFH4 (from the Institut Pasteur Collection), and ZIKV FG15 strain (provided by Dr. D. Rousset, Institut Pasteur, Cayenne). All viruses were grown using the Aedes Albopictus mosquito cell line C6/36 cultured in Leibovitz’s L-15 medium supplemented with 10% fetal bovine serum containing 0.1 mM nonessential amino acids and 1 × tryptose phosphate broth. Vero-E6 and DC-SIGN-expressing U937 cells were kindly provided by Dr M. Flamand and Dr B. Jacquelin (Institut Pasteur, Paris), respectively.

### 2.4. HLA Typing

Genomic DNA isolated from PBMCs of the study subjects by standard techniques (QIAmp, Qiagen, Hilden, Germany) was used for HLA typing. High-resolution Luminex-based typing for HLA class I and class II molecules (alleles A, B, C, and DRB1, respectively) was used according to the manufacturer’s protocol (Sequence-Specific Oligonucleotides (SSO) typing; Immucor, Inc., Peachtree City, GA, USA).

### 2.5. Serology

ZIKV seropositivity was determined using a recombinant antigen-based (ZEDIII antigen) indirect ELISA, as previously described [35]. Briefly, 96-well plates (Nunc, Life Technologies, Rochester, NY) were coated overnight at 4 °C with 50 ng of antigen in PBS. After washing, 200 μL PBS containing 3% skimmed milk and 0.1% Tween-20 were added for 1 h at 37°. The blocking solution was replaced by 100 μL of plasma diluted 1:500 in PBS containing 1.5% BSA and 0.1% Tween-20, and plates were incubated at 37 °C for 60 min. After 3 washes, bound antibodies were detected with a horseradish peroxidase-conjugated goat antihuman IgG immunoglobulin (Rockland Immunochemicals Inc. Limerick, PA, USA). Following incubation at 37 °C for 1 h and 3 washes, 100 μL of a substrate solution containing TMB (KPL SeraCare, Milford, MA, USA) was added. After 15 min incubation, the optical density (OD) was determined at 650 nm with an automated plate reader (Tecan infinite 200 pro). Each plasma sample was tested in duplicate. Plasma samples, obtained from individuals with positive DENV IgG serology collected before the ZIKV outbreak, were used as negative controls. The cut-off was calculated from the negative controls and was 0.196. DENV seropositivity was determined by indirect ELISA for IgGs (Panbio; Alere Inc., IL, USA), and by capture ELISA for IgM (Tecnosuma, Havana, Cuba) following the manufacturer’s instructions. The quantification of neutralizing and enhancing activities of antibodies against DENV and ZIKV infections was determined using a flow cytometry-based assay, as described previously [36,37]. Briefly, 10-fold serial dilutions of plasma samples were incubated at 37 °C for 1 h with either a dilution of virus inducing 7–15% of infection (for neutralization assay) or a dilution of virus inducing between 0.5% and 2% infection (for Antibody-Dependent Enhancement (ADE) Assay). Virus–antibody mixture was then added to 5 × 10^4^ cells (U937-DC-SIGN cells for neutralization of DENV1-4 infection, Vero cells for neutralization of ZIKV infection, or K562 cells for the ADE assay), for 2 h at 37 °C, after which cells were washed 2 times with fresh medium and then incubated for 24 h. The cells were then fixed with 4% paraformaldehyde, stained with 4G2 antibody conjugated to Alexa Fluor™ 488, and the percentage of infected cells was measured by flow cytometry. The neutralization titer of antibodies was expressed as the reciprocal dilution of plasma at which 50% of the virus was inhibited. Plasma samples from donors collected before ZIKV outbreak or from negative samples provided from the Kits to detect anti-DENV antibodies did not reveal any neutralization activity against ZIKV or DENV infection, respectively. For the ADE assay, the peak titer was expressed as the logarithm of reciprocal dilution of plasma at which the percentage of infection was maximal. Following the ELISA and neutralization assays, from the 71 plasma samples selected for this study, a total of 9 samples from ZIKV-immune and DENV-naïve individuals and 11 samples from DENV- and ZIKV-immune (DENV/ZIKV-immune) individuals were further selected for ELISPOT analysis.

### 2.6. RT-PCR Assays for Detection of DENV and ZIKV

RNA was extracted from plasma using the QIAamp Viral RNA Mini kit (Qiagen, Hilden, Germany) according to the manufacturer’s instructions. Samples were tested for DENV and ZIKV using a specific nested-PCR assay, as previously described [38]. Detection of ZIKV was confirmed in 3 out of the 9 plasma samples from ZIKV-immune DENV-naïve donors and in 6 out of the 11 plasma samples from DENV/ZIKV-immune donors, whereas DENV was detected in 2 out of the 11 plasma samples from DENV/ZIKV-immune donors (Table 1).

### 2.7. Viral Sequences

The identical amino acid sequence of 2 Zika viruses from Colombia (KX087102 and KU820897) was used as a reference for the set of overlapping 15-mer peptides. A total of 50 full-length protein-coding DENV sequences from Colombia (serotype 1: 14 sequences; serotype 2: 16 sequences; serotype 3: 13 sequences; serotype 4: 7 sequences) were retrieved from GenBank and used for pairwise sequence identity comparisons.

### 2.8. Peptides

All peptides were synthesized by Mimotopes (Victoria, Australia). A total of 853 15-mer peptides overlapping by 11 amino acids and 197 9-mer peptides overlapping by 8 amino acids were tested by ELISPOT assay. For the identification of T-cell epitopes, 15-mer peptides were combined into pools of 12 peptides, and individual peptides from the positive pools were tested in a second ELISPOT assay. Following the identification of the positive 15-mer peptides, and according to their HLA class I or class II restriction potential (predicted or shared between at least two donors), 9-mer peptides were synthesized and tested individually.

### 2.9. Ex Vivo IFN-γ ELISPOT Assay

PBMCs (2 × 10^5^) were incubated in 96-well flat-bottom plates (MSIPS 4510, Millipore, Millipore, Burlington, MA, USA) coated with anti-IFN-γ mAb (clone 1-D1K, Mabtech, Stockholm, Sweden) with 0.2 mL of complete RPMI containing 10% human AB serum with pools of 12 peptides (2 g/mL, final concentration) or individual peptides (1 g/mL final concentration) for 20 h. Following a 20 h incubation at 37 °C, the wells were washed with PBS/0.05% Tween 20 and then incubated with biotinylated anti-IFN-γ mAb (clone 7-B61-, Mabtech, Stockholm, Sweden) for 1 h 30 min. The spots were developed using streptavidin-alkaline phosphatase (Mabtech, Stockholm, Sweden) and BCIP/NBT substrate (Promega, Madison, WI, USA) and counted using an automated ELISPOT reader (Immunospot, Cellular Technology Limited, Cleveland, OH, USA). The number of IFN-γ-producing cells was expressed as spot-forming cells (SFC) relative to 1 × 10^6^ PBMCs. Values were calculated by subtracting the number of spots detected in the nonstimulated control wells. Values were considered positive if they were equal to or greater than 20 spots and at least three times above the means of the unstimulated control wells. As a positive control, cells were stimulated with CEF peptide pool (Mabtech, Stockholm, Sweden).

### 2.10. Immunogenicity and HLA Restrictions Prediction

The evaluation of binding possibilities of peptides to MHC class I and class II alleles was analyzed using the NetMHCpan3.0 and NetMHCIIpan3.1 servers, respectively [39,40].

### 2.11. Statistics

All data were analyzed with Prism software version 7.0 (GraphPad Software Inc., La Jolla, CA, USA). Statistical significance was determined using the nonparametric two-tailed Mann–Whitney test to compare two independent groups. Differences were considered significant at *p* < 0.05.

## 3. Results

### 3.1. Identification of Immunodominant Regions of the ZIKV Proteome

To investigate T-cell immunity induced after ZIKV infection, we examined responses from blood donors living in a ZIKV-endemic area in gamma interferon (IFN-γ)-specific ELISPOT assays. Blood samples from all study participants were first tested for the presence of Zika virus IgG, and dengue virus IgM and IgG by ELISA, and for the presence of virus-specific antibodies by flow cytometry-based neutralization assay against ZIKV and the four DENV serotypes. Recent infection with ZIKV was confirmed by RT-PCR in three out of nine plasma samples from ZIKV-immune DENV-naïve donors, and in six out of 11 plasma samples from DENV/ZIKV-immune donors, whereas recent infection with DENV was detected in two out of 11 plasma samples from DENV/ZIKV-immune donors (Table 1).

**Figure 1:**
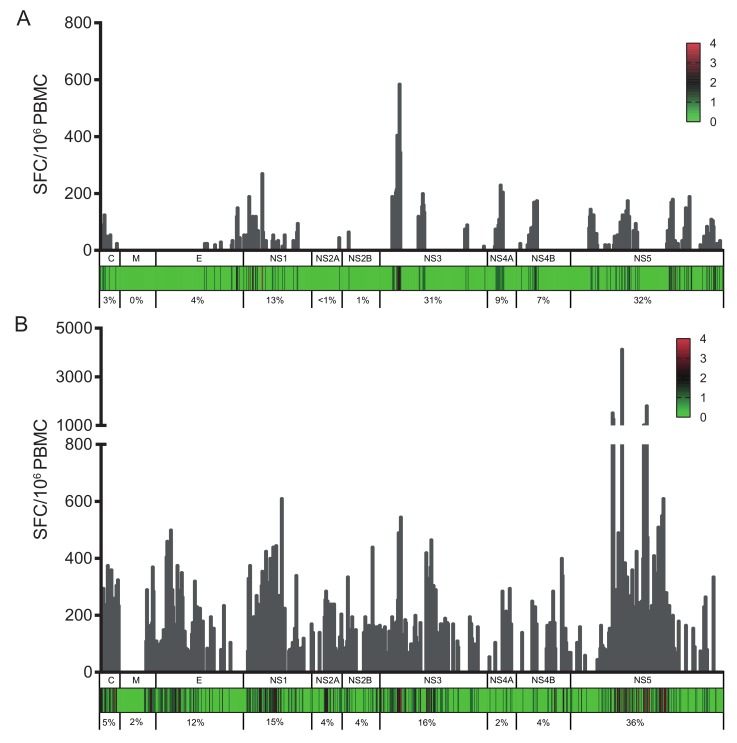
Cumulative IFN-γ responses (as spot-forming cells (SFCs) per million cells) for each overlapping peptide spanning the ZIKV proteome. (**A**) SFCs per million cells are shown for donors from group B; (**B**) SFCs per million cells are shown for donors from groups C, D, and E. The heat map indicates the number of donors with a positive IFN-γ response to each peptide within each protein (C, capsid; M, membrane; E, envelope, NS1, NS2A, NS2B, NS3, NS4A, NS4B, and NS5). The numbers below each graph represent percentages of the total response for each protein.

To confirm previous DENV or ZIKV infection in the PCR-negative samples, all samples were screened for specific neutralizing antibodies against the four DENV serotypes and against ZIKV. While all samples from ZIKV-immune DENV-naïve donors (donors from group B) revealed significant levels of neutralizing antibodies against ZIKV, they did not neutralize DENV1-4 infection, confirming the absence of DENV-specific antibodies in these donors (Figure S1). On the contrary, all samples from DENV/ZIKV-immune donors (donors from groups C, D, and E) revealed high neutralization titers against both ZIKV and DENV (Figure S1). Because anti-DENV antibodies have been shown to cross-react with ZIKV [18,20], blood samples collected before the ZIKV outbreak were used as negative controls for ZIKV neutralization. In these samples (group A donors), strong neutralization activity was measured against at least one DENV serotype, whereas low neutralization activity was detected against ZIKV, in contrast to samples from DENV/ZIKV-immune (groups C, D, and E) donors showing high neutralization activity against both DENV and ZIKV (Figure S1). Based on these data, two groups of donors were selected for further studies, donors from group B having only high levels of ZIKV-neutralizing antibodies, and donors from groups C, D, and E having both DENV- and ZIKV-neutralizing antibodies. PBMCs from all 20 ZIKV-seropositive donors (groups B to E) were screened for T-cell reactivity against pools of 15-mer peptides (overlapping by 11 amino acids) spanning the entire ZIKV proteome. Analysis of the response magnitude (as SFC per 10^6^ cells) and frequency of responding donors revealed that the nonstructural proteins NS1, NS3, and NS5 were the most vigorously and frequently recognized proteins, and accounted for more than 65% of the total response (Figure 1). As these donors were selected in DENV- and ZIKV-endemic areas, and as these viruses share an overall 43% protein-sequence identity (with up to 68% for the nonstructural proteins), we sought to distinguish between the ZIKV-specific epitopes and those shared by both viruses. Among the 20 ZIKV-seropositive blood donors, 11 individuals had both anti-DENV and anti-ZIKV IgG antibodies (C, D, and E donors) and nine individuals did not reveal any detectable anti-DENV antibodies (B donors) (Table 1 and Figure S1). Strikingly, NS1, NS3, and NS5 proteins represented 13%, 31%, and 32% of the total response, respectively, in donors from group B (Figure 1A) and 15%, 16%, and 36% of the responses in donors from groups C, D, and E (Figure 1B), whereas the NS3, NS4B, and NS5 proteins have been reported to account for 31%, 15%, and 22% of the DENV-specific T-cell response, respectively [25,41,42,43].

The analysis of the T-cell responses in these two groups of donors confirmed that NS1, NS3, and NS5 are the main targets for T cells in ZIKV-infected donors, regardless of a previous infection with DENV. From the 853 peptides spanning the entire ZIKV proteome, 410 peptides elicited a significant T-cell response, some of which being recognized by multiple donors. For most antigenic peptides, the HLA class I and class II alleles of the responding donors coincide with the alleles predicted to bind to this epitope [39,40]. Among the epitopes inducing a strong response in ZIKV-immune only and DENV/ZIKV-immune donors, several 15-mer peptides contained sequences predicted to bind strongly to at least one allele expressed by the responding donors (Table 2 and Table S1).

For instance, the NS2B_1171–31_ peptide contains a 10-mer sequence predicted to bind strongly to the HLA-A*03:01 and -A*11:01 molecules expressed by the responding donor 55 (Table S1). In other cases, multiple responding donors express at least one common allele with strong potential for binding to the stimulating peptide. This holds for the E_4554–69_ peptide in the envelope that contains the 9-mer and the 10-mer sequences predicted to bind to the HLA-B*51:01 and HLA-A*02:01 alleles, both alleles being expressed by the responding donors 1 and 77 (Table S1). This also applies to the NS5_132–7_ peptide, which induced a strong response in donors 55 and 69 that share the HLA-B*35:01 allele, this allele being predicted to bind to the 9-mer peptide MSALEFYSY with a high affinity (Table S1). Interestingly, this epitope was also shown to induce a significant response in transgenic mice carrying the HLA-A*01:01 molecule, which is expressed by donor 69 [23]. Similarly, a strong T-cell response was observed against the NS5_5465–60_ peptide in donors 28, 53, and 66 that express the HLA-B*40:02 and -B*44:03 alleles, and against the NS5_6056–19_ peptide in donors 33 and 59 that share the predicted HLA-A*24:02 allele (Table S1). Finally, we have also identified several 9-mer and 15-mer immunodominant epitopes in the Capsid, NS1, NS3, NS4B, and NS5 proteins, which induced substantial T-cell responses in donors that share one or several alleles with a strong potential for binding to these peptides (Table 2).

Within the NS3 and NS5 proteins, several epitopes were previously described as immunodominant epitopes, either predicted or validated experimentally after DENV infection or vaccination in humans or after ZIKV infection in mice and in humans [22,23,29,44,45]. Indeed, among the 9-mer peptides identified in DENV/ZIKV-immune (groups C, D, and E) donors, the NS5_2933–07_, NS5_2973–11_, and NS5_3453–59_ have been already detected in PBMCs from HLA-B*35:01 individuals after infection with DENV1, DENV2, or vaccination with DENV live attenuated vaccine (DLAV), with a lysine-to-arginine and a phenylalanine-to-tyrosine amino acid substitution at residues 302 and 350 in the NS5_2973–11_ and NS5_3453–59_ peptides from ZIKV, respectively [22,43,44,46] (Table 2). These results thus confirm that these NS5 peptides contain nested epitopes restricted by the HLA-B*35:01 molecule. Yet the 15-mer NS3_2192–33_ peptide, which contains the APTRVVAAEM epitope, induced a substantial response in two donors with a history of ZIKV and DENV infection that express neither HLA-B*07:02 nor B*35:01 (Table S1), although these alleles were expressed in responding donors vaccinated with the live attenuated DENV vaccine DLAV or in *ifnar*-/- HLA-B*07:02 transgenic mice or in humans after ZIKV infection [22,23,44]. This suggests that the NS3_2192–33_ peptide contains another epitope or a promiscuous epitope that binds to other HLA alleles besides HLA-B*07:02 or B*35:01 (Table 2).

### 3.2. Broader Responses with a Higher Magnitude in DENV/ZIKV-Immune Donors

**Figure 2:**
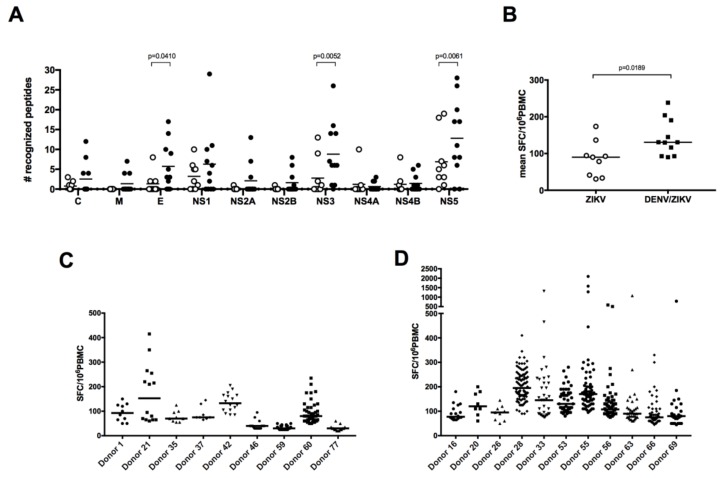
DENV/ZIKV-immune donors reveal a broader T-cell response with a higher magnitude. (**A**) Breadth and (**B**) magnitude of responses in donors with a history of ZIKV infection only or DENV and ZIKV infection. Each dot represents one donor (open circles, ZIKV-immune DENV-naïve donors; filled circles, DENV/ZIKV-immune donors) and the bars represent the median value for each group of donors. The *p* values were calculated using the nonparametric two-tailed Mann–Whitney test. Frequency of responses against individual peptides, per donor, in ZIKV-immune DENV-naïve (**C**) and DENV/ZIKV-immune (**D**) donors. Each dot represents one peptide. The bars represent the median response for each donor.

Given the ZIKV-specific antibody response against NS1 and the low level of CD4 T-cell cross-reactivity between DENV and ZIKV against the E and NS1 proteins [18], we wished to compare, among the immunodominant epitopes, the T-cell responses in PBMCs from ZIKV-immune only (group B) donors with those from DENV/ZIKV-immune (groups C, D, and E) donors. First, comparison of the frequency of responding T cells between these two different types of donors underlined the higher magnitude of response in donors with evidence of past or current ZIKV and DENV infection relative to donors with a history of ZIKV infection only (Figure 1A,B). The number of stimulating peptides per donor for each viral protein, as well as the average response per donor differed in these two groups, with a significantly broader response against the E, NS3, and NS5 proteins and a higher magnitude of response in DENV/ZIKV-immune (Figure 2A,B). To determine whether this difference concerned only a small number of peptides that elicit a stronger response in each donor, or if it concerned the majority of the peptides, we plotted the frequency of responses against the different peptides, per donor, in the two different groups. Two out of nine individuals among the ZIKV-immune DENV-naïve (group B) donors revealed a median response higher than 100 SFC/million cells, whereas six out of 11 DENV/ZIKV-immune (groups C, D, and E) donors developed this strong response, which was also directed against a higher number of peptides (Figure 2C,D). This result reveals the activation of a higher frequency of T cells against ZIKV peptides, with a higher magnitude of response, in donors previously or currently infected with DENV, in comparison with naïve donors. This strongly argues for the existence of cross-reactive T cells, these T cells being primed during the initial infection with DENV and expanded thereafter during the following infection with ZIKV, as shown recently in mice and in humans, after sequential infection with DENV and ZIKV [22,23].

### 3.3. DENV/ZIKV-Cross-Reactive T Cells Mainly Target the NS5 Protein

To identify, more specifically, ZIKV-specific peptides and DENV/ZIKV cross-reactive peptides, we compared the sequences of the most immunodominant epitopes recognized by both types of donors. The NS1 and NS3 proteins contain a high proportion of peptides that elicit strong responses in both ZIKV-immune DENV-naïve and DENV/ZIKV-immune donors, whereas the E protein and to a higher extent the NS5 protein contain a majority of peptides inducing a strong response only in DENV/ZIKV-immune donors (Figure 2A and Table 3). This suggests that the NS1 and NS3 proteins contain more ZIKV-specific epitopes, whereas the NS5 protein contains more epitopes shared by DENV and ZIKV and recognized by cross-reactive T cells. Strikingly, most of the peptides recognized only by DENV/ZIKV-immune donors exhibit high degree of identity with the four DENV serotypes. For instance, in the NS1 protein, two out of the five epitopes that induced a response in ZIKV-immune DENV-naïve donors revealed a sequence identity higher than 60% with the four DENV serotypes, whereas eight out of the 11 epitopes in the NS5 protein that induced a strong response in DENV/ZIKV-immune donors showed a sequence identity higher than 67% with the four DENV serotypes (Table 3). Altogether, these data strongly support the activation of cross-reactive T cells induced after DENV and ZIKV infection, which recognize common epitopes between DENV and ZIKV mainly located in the NS5 protein, and which dominate the T-cell response against ZIKV.

### 3.4. Increased Neutralizing Antibody Titer against ZIKV in DENV/ZIKV-Immune Donors

**Figure 3:**
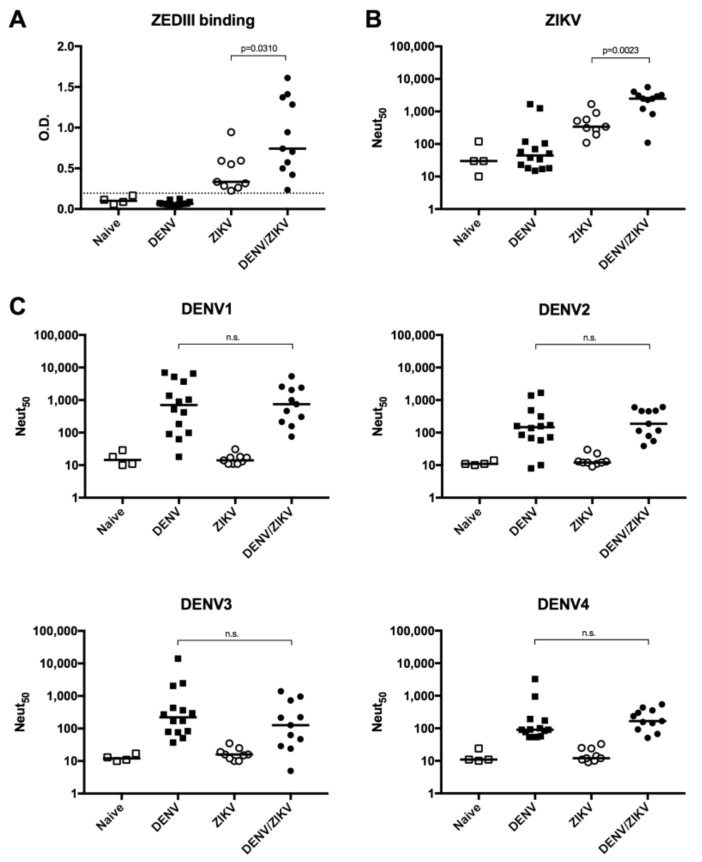
Analysis of ZIKV EDIII (ZEDIII)-binding and ZIKV- and DENV-neutralizing activity of plasma samples from DENV- and/or ZIKV-immune donors. (**A**) Detection of ZEDIII-binding antibodies by ELISA; (**B**) quantification of neutralizing activity against ZIKV; (**C**) quantification of neutralizing activity against DENV1, 2, 3, and 4. Naïve: plasma samples from naïve donors; DENV: plasma samples from DENV-immune ZIKV-naïve donors; ZIKV: plasma samples from ZIKV-immune DENV-naïve donors; DENV/ZIKV: plasma samples from DENV-immune ZIKV-immune donors. Each dot represents one donor and the bars represent the median value for each group of donors. The *p* values were calculated using the nonparametric two-tailed Mann–Whitney test; n.s.: not significant. The dotted line represents the ELISA cut-off value.

To determine whether the high T-cell response in DENV/ZIKV-immune donors is also accompanied by a strong antibody response against ZIKV in these donors, we analyzed the ability of ZIKV-specific antibodies to bind and to neutralize ZIKV and DENV1-4. Plasma samples from ZIKV-immune DENV-naïve (group B) donors, DENV/ZIKV-immune (groups C, D, and E) donors, as well as DENV-immune ZIKV-naïve (group A) donors were used to analyze the ability of antibodies to bind to ZIKV EDIII (ZEDIII) and to neutralize ZIKV infection. Results revealed an increased level of antibodies that bind to ZEDIII in plasma samples from ZIKV-immune DENV-immune donors, in comparison with plasma samples from ZIKV-immune DENV-naïve donors (Figure 3A). On the contrary, plasma samples from DENV-immune ZIKV-naïve donors did not reveal any binding activity on ZEDIII.

Knowing that EDIII-specific antibodies are more efficient in neutralizing ZIKV infection [47], we then asked whether the higher binding capacity to ZEDIII is also accompanied by a stronger neutralization against ZIKV infection. Comparison between the neutralization activity of antibodies between ZIKV-immune DENV-naïve (group B) donors and DENV/ZIKV-immune (groups C, D, and E) donors revealed a significantly higher neutralization potential against ZIKV in donors with evidence of past or current ZIKV and DENV infection (Figure 3B). Interestingly, while most DENV-immune ZIKV-naïve donors did not show any neutralization against ZIKV infection, two out of 14 DENV-immune donors in this group could also neutralize ZIKV (Figure 3B and Figure S1), even though these two plasma samples were collected before the ZIKV outbreak, confirming the induction of ZIKV-cross-neutralizing and non-EDIII-binding antibodies in DENV-immune donors [18,20,48].

To determine whether these neutralizing antibodies against ZIKV infection could also cross-neutralize DENV, we evaluated the ability of these antibodies to neutralize the 4 DENV serotypes. Comparison of the neutralizing activity of samples from DENV-immune ZIKV-naïve and DENV/ZIKV-immune donors did not reveal any increase in the neutralization titer against the four DENV serotypes, showing that a subsequent ZIKV infection in DENV-immune donors only increases the level of ZIKV-neutralizing antibodies without affecting the level of DENV-neutralizing antibodies (Figure 3C). In addition, no DENV-neutralizing activity was detected in ZIKV donors. These data confirm previous studies on the induction of ZIKV-specific neutralizing antibodies induced after ZIKV infection independently of prior DENV immunity [48].

### 3.5. Decreased Enhancing Potential on ZIKV Infection in DENV/ZIKV-Immune Donors

**Figure 4:**
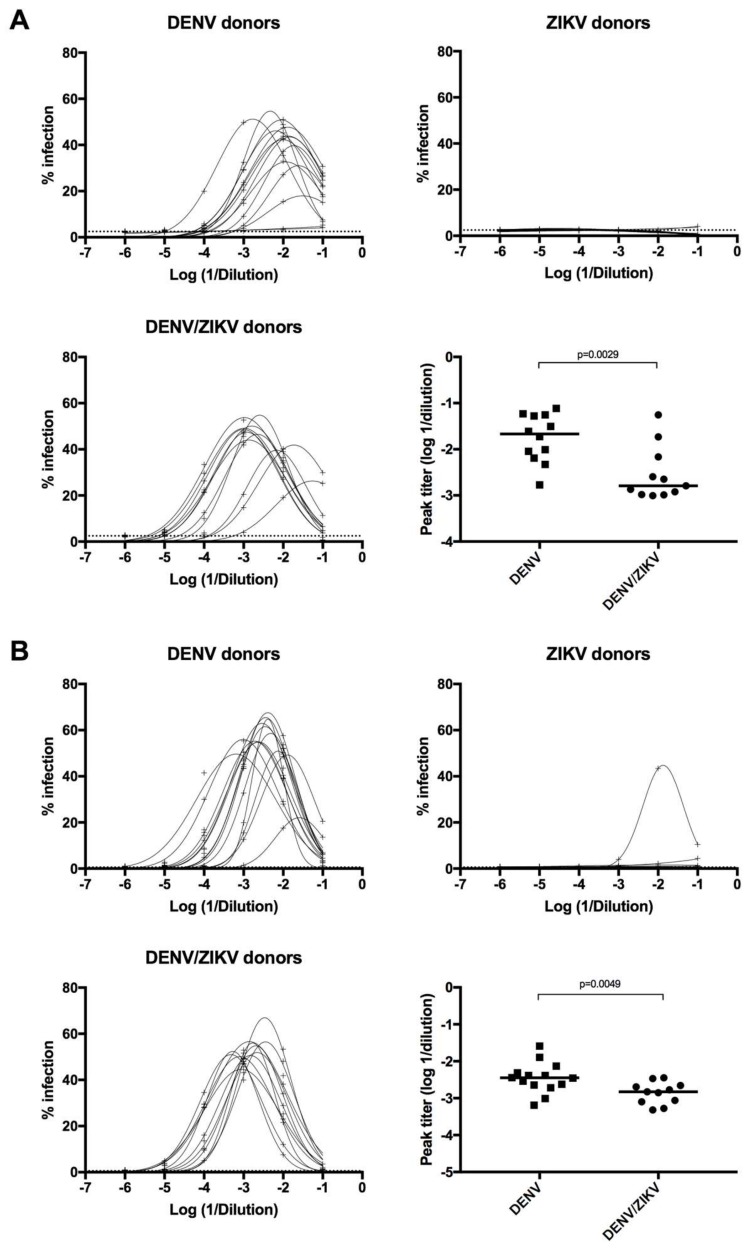
Analysis of Antibody-Dependent Enhancement (ADE) activity of plasma samples on ZIKV and DENV4 infection. (**A**) Detection of ADE activity on ZIKV infection from plasma samples of DENV-immune ZIKV-naïve (higher-left panel), ZIKV-immune DENV-naïve (higher-right panel), and DENV/ZIKV-immune (lower-left panel) donors. Comparison of the peak enhancement titer of antibodies from DENV-immune ZIKV-naïve and DENV/ZIKV-immune donors (lower-right panel). Each dot represents one donor; (**B**) detection of ADE activity on DENV4 infection from plasma samples of DENV-immune ZIKV-naïve (higher-left panel), ZIKV-immune DENV-naïve (higher-right panel), and DENV/ZIKV-immune (lower-left panel) donors. Infectivity curves for each donor are shown. The *p* values were calculated using the nonparametric two-tailed Mann–Whitney test. The dotted line represents the ELISA cut-off value.

As DENV infection was previously shown to induce subneutralizing antibodies against DENV1-4 and ZIKV, which could mediate enhancement of DENV and ZIKV infection [17,18,20,21,49,50], we measured the ADE activity of plasma samples from DENV-immune ZIKV-naïve donors, ZIKV-immune DENV-naïve donors, and DENV/ZIKV-immune donors on ZIKV infection and DENV1-4 infection in vitro. Comparison of the peak enhancement titer between these different samples showed that plasma samples from DENV-immune ZIKV-naïve (group A) donors have an enhancing potential on ZIKV infection, whereas plasma samples from ZIKV-immune DENV-naïve (group B) donors do not have this ADE activity (Figure 4A).

More strikingly, a significant decrease in the ADE activity for ZIKV infection was observed in DENV/ZIKV-immune samples, in comparison with DENV-immune ZIKV-naïve samples (Figure 4A). These results clearly show that the ADE activity against ZIKV infection, induced after a DENV infection, decreases after a subsequent infection with ZIKV. Interestingly, this decrease in the ADE activity was also observed, albeit to a lesser extent, against DENV4 infection, but not against DENV1, 2, and 3 (Figure 4B). These results highlight the ability of a subsequent ZIKV infection to reduce the enhancing activity against a DENV4 strain in vitro but not against the other DENV serotypes. Analyses of the ADE activity using a higher number of DENV-immune ZIKV-naïve and DENV/ZIKV-immune donors should confirm these results. Interestingly, one out of nine samples from ZIKV-immune DENV-naïve donors revealed a clear ADE activity on DENV4 infection but not on DENV1-3 infection (Figure 4B). These results show that ZIKV infection in DENV-naïve donors can induce antibodies with enhancing activity against DENV4 in some donors, thus confirming previous reports showing the ability of ZIKV-induced antibodies to enhance DENV infection in vitro [18]. They also show that the ADE activity against DENV4 is reduced following sequential DENV and ZIKV infection.

### 3.6. High Titer of Neutralizing Antibodies with Low Enhancing Activity are Associated with Strong T-Cell Responses

**Figure 5:**
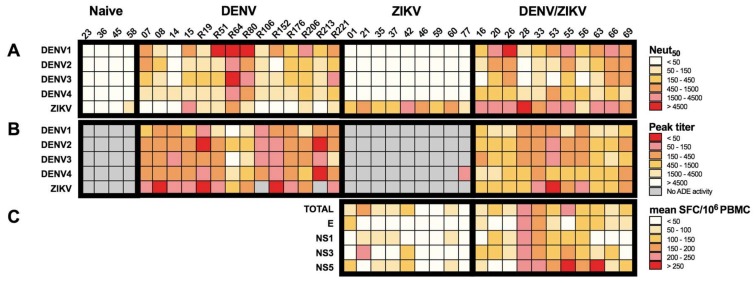
Specificity and cross-reactivity of antibody and T-cell responses from donors with DENV and/or ZIKV infection. (**A**) Heat map of the neutralizing activity of plasma samples from DENV-immune ZIKV-naïve, ZIKV-immune DENV-naïve, and DEN/ZIKV-immune donors on DENV1-4 and ZIKV infection. (**B**) Heat map of the ADE activity of plasma samples from DENV-immune ZIKV-naïve, ZIKV-immune DENV-naïve, and DENV/ZIKV-immune donors on DENV1-4 and ZIKV infection; (**C**) Heat map of the mean T-cell response against peptides from the whole ZIKV proteome (TOTAL) or the immunodominant proteins of PBMCs from ZIKV-immune DENV-naïve and DENV/ZIKV-immune donors; each column represents one donor.

Considering that DENV/ZIKV-immune donors have a higher titer of neutralizing antibodies against ZIKV in comparison with ZIKV-immune DENV-naïve donors, we wondered whether a significant correlation could be established between the level of neutralizing antibodies and the strength of the T-cell response. Although no significant correlation could be drawn between these two different parameters of the immune response, the analysis of T- and B-cell responses in individual donors revealed a higher number of DENV/ZIKV-immune donors having both a high neutralizing antibody response (and low ADE activity) and a strong cross-reactive T-cell response against ZIKV than ZIKV-immune DENV-naïve donors (Figure 5).

For instance, six out of eight DENV/ZIKV-immune donors with a Neut_50_ value higher than 1500 revealed a moderate/low ADE activity (peak titer ≥ 450) on ZIKV infection and a moderate/high T-cell response (mean SFC/10^6^ PBMC ≥ 100) against E, NS1, NS3, or NS5 (donors 16, 20, 28, 55, 63, and 66). On the contrary, donors with a moderate/low neutralizing activity against ZIKV (donors 33, 56, and 69, Neut_50_ ≤ 1500) revealed a moderate/high ZIKV ADE activity (peak titer ≤ 450) and variable T-cell responses against E, NS1, NS3, or NS5 proteins (Figure 5). Conversely, only one out of nine ZIKV-immune DENV-naïve donors revealed high neutralizing activity against ZIKV, with moderate and low T-cell activity against NS3 and NS5, and against E and NS1, respectively. With the exception of one donor (donor 21) showing high T-cell activity against NS3 and a moderate/low level of neutralizing antibodies, all the other ZIKV-immune DENV-naïve donors exhibited low T-cell activity and a moderate/low level of neutralizing antibodies. These results suggest that cross-reactive T cells, which are induced after a sequential DENV and ZIKV infection, and exhibit strong functional activity against specific ZIKV epitopes, could play a role in the induction of antibodies with high neutralizing potential against ZIKV.

## 4. Discussion

In this study, using PBMCs from ZIKV-infected human blood donors, we have identified numerous T-cell epitopes specific to ZIKV or shared between DENV and ZIKV. While the DENV-specific T-cell responses are predominantly directed against NS3, NS4B, and NS5, the response against ZIKV mainly targets epitopes in the NS1, NS3, and NS5 proteins. The stronger and broader IFN-γ response against peptides from the NS5 protein observed in donors previously infected with DENV led us to postulate that this region contains more peptides recognized by cross-reactive T cells, whereas the NS1 protein is preferentially targeted by ZIKV-specific T cells, which is consistent with the higher percentage of identity observed between ZIKV and DENV sequences in the NS5 protein, in comparison with the NS1 protein.

Among the epitopes activating cross-reactive T cells, several peptides in the NS5 protein matched the T-cell epitopes recently identified from ZIKV-positive donors, such as the NS5_2983–06_, NS5_3483–56_, and NS5_4614–75_ peptides recognized by CD8 and CD4 T cells, in the context of HLA-B*35:01 and HLA-DRB1*07:01, respectively [22]. For several epitopes, the 15-mer or 9-mer peptides matched epitopes recently identified in transgenic mice expressing human HLA molecules, or in humans exposed to ZIKV, thus confirming the class I allele restriction for these peptides. This is the case for 15-mer peptide VARVSPFGGLKRLPA inducing a response in a donor expressing the HLA-B*07:02 allele, which contains the C_253–5_ peptide SPFGGLKRLPA shown to elicit a significant response in HLA-B*07:02 transgenic mice infected with ZIKV [23]. The same correlations have been established with NS3 (FPDSNSPIM), NS4B (RGSYLAGASLIYTVT), and NS5 (NQMSALEFYSY) peptides that induced a strong response in human donors expressing the HLA-B*07:02 and HLA-A*01:01 alleles, respectively (Table S1), and in transgenic mice expressing these alleles [23]. In other cases, the epitopes identified in HLA-B*07:02 and HLA-A*01:01 transgenic mice were also identified in responding donors that nevertheless do not express these alleles, such as the NS3_2192–33_ peptide (Table 2), and the NS1_193–3_ or the NS5_132–7_ peptides (Table 3 and Table S1), which elicit a response in donors that express neither of the two alleles, HLA-B*07:02 or HLA-A*01:01. For these donors, one possibility could be that the epitope identified in transgenic mice has a higher affinity for a human HLA allele different from the allele expressed by the transgenic mice, or that the 15-mer peptide contains another epitope that binds to a different allele. Binding studies with 9-mer epitopes and HLA class I stabilization assays using TAP-deficient cells should discriminate between these possibilities.

We also reported the identification of several peptides that share common sequences with DENV and are preferentially targeted by cross-reactive T cells, after DENV and ZIKV infection. Among these peptides, the NS5_2933–07_ and NS5_2973–11_ peptides contain the amino acid sequence HPYRTWAYH that shares seven amino acids with an epitope identified in DENV1-positive or in DENV-positive and ZIKV-positive donors [22,46]. Similarly, the NS5_3253–39_ peptide contains the amino acid sequence KPWDVVTGV, which is 67% identical to the epitope KPWDVIPMV identified in individuals infected with DENV1 [46]. Finally, the strongest T-cell responses in DENV/ZIKV-immune donors were observed with the NS5_4814–95_ peptide or the NS5_3453–59_ and the NS5_4654–79_ peptides (Table 3), which contain 9-mer epitopes identified previously in DENV-infected individuals [44] or, more recently, in ZIKV-positive donors, respectively [22]. Altogether, these data reveal the activation of DENV/ZIKV cross-reactive T cells that dominate the response following sequential DENV and ZIKV infection. Notably, although these cross-reactive peptides exhibit a high degree of sequence identity with DENV and can stimulate a T-cell response after DENV infection, they do not induce a response after primary infection with ZIKV, suggesting that they are immunodominant in the context of DENV but not in the context of ZIKV infection. This result is expected, as the immunodominance of an epitope or its relative abundance depends on the other epitopes expressed by the protein. This is also in agreement with previous observations showing that epitope production correlates with cleavability of flanking residues expressed in the protein sequence [51].

Importantly, for these cross-reactive epitopes, the absence of a T-cell response in ZIKV-infected donors is not simply due to the absence of the presenting HLA allele in this population, as most of the alleles expressed in responding DENV/ZIKV-immune donors were also expressed in ZIKV-immune DENV-naïve donors (Table S1). This is what we observed for the NS5_132–7_, NS5_2933–07_, NS5_3453–59_, and NS5_5465–60_ epitopes, predicted to be strong binders to the HLA-B*35:01 and HLA-B*40:02 alleles, respectively, that were frequently expressed by our ZIKV-immune DENV-naïve donors (Table S1). Altogether, these results show that, in the case of initial ZIKV infection, there is a preferential recognition of ZIKV-specific epitopes, whereas there is a more frequent and stronger T-cell response against cross-reactive epitopes after sequential heterologous DENV/ZIKV infection. Interestingly, the strong T-cell response observed in DENV/ZIKV-immune donors against these NS5 epitopes relies primarily on donors that express the HLA-B*3501 allele, an allele associated with high-magnitude responses against DENV, and a stronger protection against DENV infection and disease [25]. As all blood samples were obtained from donors with asymptomatic ZIKV infection history, we cannot relate the strength of the ZIKV-specific T-cell response obtained in HLA-B*35:01 donors to the protection against the disease. Further studies with more subjects with a higher susceptibility to disease following primary ZIKV infection are required to determine whether, as for DENV, there is an HLA-linked protective role for T cells in ZIKV infection. Likewise, it would also be important to compare disease severity in donors having experienced a previous DENV infection or not, to determine whether cross-reactive T cells induced after DENV infection could mediate a better protection against ZIKV infection and disease, as recently suggested in mice [23,31]. As both CD4+ and CD8+ T cells were shown to contribute to protection against DENV infection, a comprehensive analysis of MHC class II-restricted response is needed to determine the role of CD4 in ZIKV infection and disease protection. Finally, further phenotypic analyses of ZIKV-specific T cells in asymptomatic or symptomatic donors will help in defining correlates of protection in natural immunity and vaccination against ZIKV infection and disease. It will be particularly important to determine whether, as for DENV-specific T cells, strong responses against ZIKV-specific peptides are more frequent for specific HLA alleles and are associated with multifunctionality [25].

From the same individuals, we have also shown that, while a primary DENV infection induces cross-reactive antibodies with enhancing activity against the heterologous DENV serotypes and against ZIKV, a primary ZIKV infection in naïve donors mainly elicits ZIKV-specific antibodies with no enhancing activity against DENV. These results do not coincide with the enhancing activity described in the plasma of one donor infected with ZIKV only [52]. Apart from the fact that the ADE activity detected in vitro could result from the presence of cross-reactive antibodies induced not exclusively after DENV or ZIKV infection, this discrepancy could be due to the too-low number of ZIKV-immune DENV-naïve donors with low neutralizing-antibody titers (three donors with Neut_50_ values < 300). Alternatively, as the ADE activity in one donor was revealed from day 48 post-onset of symptoms [52], and since all donors from our cohort were asymptomatic, it is possible that the samples from ZIKV-immune donors were collected at an early time after infection, prior to the appearance of enhancing activity against ZIKV infection. In agreement with previous studies, we have also observed a higher level of cross-reactive antibodies between DENV and ZIKV in donors who have been recently infected with DENV [17,48,53], as in the case of donors 55, 63, and 66 from group C donors, donors 26 and 53 from group D donors, and donors 20 and 69 from group E donors (Figure 5). Our study also clearly reveals a significant increase in the titer of neutralizing antibodies against ZIKV infection in ZIKV-immune donors with previous or current DENV infection (donors from groups C, D, and E), in comparison with ZIKV-immune DENV-naïve donors (Figure 3B). However, while the increased titer of neutralizing antibodies between donors from B and C is still significant, we did not observe any significant difference in the titer of ZIKV-neutralizing antibodies between donors from groups B (with sequential DENV and ZIKV infections) and D (DENV-immune with acute ZIKV infection), or from groups B and E (acute DENV and ZIKV infections). A higher number of donors from groups D and E should allow us to determine whether there is also an increase in the level of ZIKV-neutralizing antibodies in donors with acute DENV and/or ZIKV infection. Strikingly, while ZIKV infection in DENV-immune donors was shown to increase the level of neutralizing antibodies against ZIKV, in comparison with DENV-naïve donors, it decreases the enhancing activity of antibodies against ZIKV, in comparison with donors infected only with DENV. These results are consistent with the inverse correlation observed between the level of neutralizing antibodies and enhancing activity in DENV-immune donors [54,55]. Interestingly, while ZIKV infection in DENV-immune donors does not modulate the enhancing potential of antibodies against DENV1-3 infection, it decreases the ADE activity against DENV4 infection in vitro, a result that could be explained by the higher amino acid sequence identity in the E protein between ZIKV and DENV4, in comparison with DENV1-3 [56]. 

Taken together, these results strongly support the activation and clonal expansion of memory B cells induced after sequential DENV and ZIKV infection, as shown recently in DENV1-infected individuals [57,58]. These data also show that, while a sequential DENV and ZIKV infection results in increased neutralization potential and decreased enhancing activity against ZIKV, it does not affect the level of neutralizing or enhancing antibodies against DENV, suggesting that ZIKV infection in DENV-immune individuals should not have a significant impact on dengue disease following subsequent DENV infection. However, as a small fraction of individuals infected with ZIKV were only shown to express antibodies with enhancing activity against DENV4, it will be important in the future to analyze in these individuals the impact of ZIKV infection on the outcome of dengue disease following DENV4 infection. In this sense, it is also worth mentioning that the modulation of ADE activity measured in vitro does not necessarily reflect the in vivo situation, given in particular the conflicting results on the impact of pre-existing immunity to DENV on ZIKV pathogenesis [21,59,60]. Finally, in light of the stronger T-cell response and the higher titer in neutralizing antibodies against ZIKV observed in individuals sequentially infected with DENV and ZIKV, it would be important to determine whether antigen-specific T follicular-helper (Tfh) cells could be detected in these individuals, which could account for the activation of memory B cells, as shown recently in PBMC of DENV-infected patients [61]. In this sense, our study provides a framework for detailed analyses of cell populations potentially linked to the activation of memory B cells with high neutralization potential against DENV and ZIKV.

## 5. Patents

C.R., E.S.-L., A.S., F.G.D. are inventors of a patent filing related to this work.

## Figures and Tables

**Table 1:** Categories of donors in the study cohort.

Group	Nb of Samples	ZIKV Status ^1,2^	DENV Status ^1,2^	Age ^3^ (Year)	Sex (% Male)
A	14	Negative	Positive	17 (84–1)	36
B	9	Positive	Negative	30 (184–1)	56
C	5	Positive	Positive	27 (213–5)	20
D	4	Acute	Positive	33 (205–4)	25
E	2	Acute	Acute	27 (252–8)	50

^1^ Previous exposure to ZIKV or DENV was determined by the presence of ZIKV- or DENV-specific IgG antibodies; negative, absence of virus-specific IgG antibodies; positive, presence of virus-specific IgG antibodies; ^2^ acute infection with ZIKV or DENV was confirmed by RT-PCR; ^3^ expressed as the mean age of the group (range). ZIKV: Zika virus; DENV: dengue virus.

**Table 2:** HLA class I- or class II-restricted T-cell epitopes identified in ZIKV proteins.

Peptide ^1^	Sequence	SFC/Million PBMC ^2^	HLA	Score ^3^
C_869–4_	KDLAAMLRI	65	B*55:01	1.4
			B*40:02	2.0
C_899–7_	AAMLRIINA	75	A*02:01	4.5
NS1_637–7_	MENIMWRSVEGELNA	310	DRB1*04:05	50
NS1_687–6_	WRSVEGELN	206	B*40:02	48
			B*18:01	48
NS1_1671–75_	VWLKVREDY	75	A*29:02	1.3
NS1_2802–88_	CPGTKVHVE	170	B*35:01	8.5
			B*35:31	6.5
NS3_2192–33_	TVILAPTRVVAAEME	165	DRB1*08:02	1.5
NS3_3113–25_	AAIFMTATPPGTRDA	470	DRB1*04:01	4.0
NS3_3133–21_	IFMTATPPG	30	A*24:02	5.0
NS3_3143–22_	FMTATPPGT	85	A*02:17	5.5
NS4B_1121–20_	AIILLVAHY	88	A*29:02	0.6
			A*11:01	3.5
NS4B_1151–23_	LLVAHYMYL	68	A*02:05	0.3
NS4B_1161–24_	LVAHYMYLI	35	A*69:01	0.15
			A*02:05	0.2
NS5_2983–06_	NHPYRTWAY ^4^	358	B*35:01	3.0
NS5_2993–07_	HPYRTWAYH ^4^	308	B*35:01	0.4
NS5_3023–10_	RTWAYHGSY	205	A*01:01	0.5
NS5_3483–56_	TPYGQQRVF ^4^	1681	B*35:31	0.7
			B*35:01	0.3
NS5_4254–33_	EAVNDPRFW	465	B*44:03	5.0
			B*15:17	7.0
			B*35:01	5.0
NS5_4614–75_	KKQGEFGKAKGSRAI	405	DRB1*07:01	32
NS5_4734–87_	RAIWYMWLGARFLEF	505	DRB1*07:01	16
NS5_6096–17_	YALNTFTNL	42	B*35:43	0.4
			B*35:31	0.25

^1^ The position of peptides were determined according to NCBI reference sequence YP_002790881.1; ^2^ Cumulative SFC/million PBMC; ^3^ Calculated using NetMHCpan 3.0 or NetMHCIIpan3.1 server; ^4^ Peptides identified previously in human individuals infected with DENV and ZIKV [22].

**Table 3:** Immunodominant epitopes in ZIKV- and DENV/ZIKV-immune donors.

Peptide ^1^	Sequence	ZIKV		DENV/ZIKV		Identity (%)			
		Donors	SFC/million PBMC ^2^	Donors	SFC/million PBMC ^2^	DENV Serotypes			
						1	2	3	4
C_49-63_	AILAFLRFTAIKPSL	60	60	28,63	365	60	53	60	40
E_67-81_	DMASDSRCPTQGEAY			33	465	67	53	67	53
E_87-101_	DTQYVCKRTLVDRGW			56	505	67	54	73	67
NS1_19-33_	VFVYNDVEAWRDRYK	21,46,60	195	28,56	380	47	33	47	40
NS1_55-69_	CGISSVSRMENIMWR	35,46	125	56	275	67	66	60	60
NS1_91-105_	GSVKNPMWRGPQRLP	21,35,46,60	275	28	165	13	33	20	33
NS1_107-121_	PVNELPHGWKAWGKS			28,53	430	40	47	47	50
NS1_147-161_	HRAWNSFLVEDHGFG	46	40	33,53	445	67	73	67	76
NS1_163-177_	FHTSVWLKVREDYSL	46	35	20,28,55	450	47	46	53	47
NS1_195-209_	HSDLGYWIESEKNDT			28,33	615	80	73	66	73
NS2B_117-131_	AAGAWYVYVKTGKRS			55	445	33	33	27	27
NS3_131-145_	PAGTSGSPILDKCGR	21,42	405	26,55,63	495	53	61	53	54
NS3_143-157_	CGRVIGLYGNGVVIK	21	350	20,55,63,66	550	60	67	72	80
NS3_311-325_	AAIFMTATPPGTRDA			28,33	470	80	80	93	80
NS5_13-27_	KARLNQMSALEFYSY			55,69	405	53	47	53	40
NS5_293-307_	WFFDENHPYRTWAYH			55,69	1620	67	67	60	67
NS5_297-311_	ENHPYRTWAYHGSYE			55,69	1330	80	80	73	80
NS5_325-339_	VVRLLSKPWDVVTGV			28,55,66	495	73	80	73	67
NS5_345-359_	TDTTPYGQQRVFKEK			33,55,69	4195	93	93	93	93
NS5_373-387_	QVMSMVSSWLWKELG	60	130	55,66,69	340	40	53	47	47
NS5_461-475_	KKQGEFGKAKGSRAI			28,53	405	93	93	93	87
NS5_465-479_	EFGKAKGSRAIWYMW			28,53,55,56	1085	100	100	100	93
NS5_473-487_	RAIWYMWLGARFLEF			28,55	505	100	100	93	100
NS5_481-495_	GARFLEFEALGFLNE			28,53,56,63	1870	93	100	93	100
NS5_546-560_	RFDLENEALITNQME			28,53,66	515	60	47	53	60
NS5_573-586_	TYQNKVVKVLRPAEK			28,53,56	615	73	67	73	80
NS5_849-863_	CGSLIGHRPRTTWAE	60	90	33,55	340	67	67	67	67

^1^ The position of peptides was determined according to NCBI Reference Sequence YP_002790881.1; ^2^ Cumulative SFC/million PBMC.

## Supplementary Materials

The following are available online at http://www.mdpi.com/1999-4915/10/9/480/s1, Figure S1: Comparison of the neutralization activity against ZIKV and DENV1-4 infection between plasma samples from all donors in the study cohort. Table S1: Characteristics of antigenic peptides from ZIKV identified in this study.

## References

[1] Kuno G., Chang G.J., Tsuchiya K.R., Karabatsos N., Cropp C.B. (1998). Phylogeny of the genus Flavivirus. J. Virol..

[2] Dick G.W., Kitchen S.F., Haddow A.J. (1952). Zika virus. I. Isolations and serological specificity. Trans. R Soc. Trop. Med. Hyg..

[3] Duffy M.R., Chen T.H., Hancock W.T., Powers A.M., Kool J.L., Lanciotti R.S., Pretrick M., Marfel M., Holzbauer S., Dubray C. (2009). Zika virus outbreak on Yap Island, Federated States of Micronesia. N. Engl. J. Med..

[4] Cao-Lormeau V.M., Roche C., Teissier A., Robin E., Berry A.L., Mallet H.P., Sall A.A., Musso D. (2014). Zika virus, French polynesia, South pacific, 2013. Emerg. Infect. Dis..

[5] Campos G.S., Bandeira A.C., Sardi S.I. (2015). Zika Virus Outbreak, Bahia, Brazil. Emerg. Infect. Dis..

[6] Dupont-Rouzeyrol M., O’Connor O., Calvez E., Daures M., John M., Grangeon J.P., Gourinat A.C. (2015). Co-infection with Zika and dengue viruses in 2 patients, New Caledonia, 2014. Emerg. Infect. Dis..

[7] Zanluca C., Melo V.C., Mosimann A.L., Santos G.I., Santos C.N., Luz K. (2015). First report of autochthonous transmission of Zika virus in Brazil. Mem. Inst. Oswaldo Cruz..

[8] Pacheco O., Beltran M., Nelson C.A., Valencia D., Tolosa N., Farr S.L., Padilla A.V., Tong V.T., Cuevas E.L., Espinosa-Bode A. (2016). Zika virus disease in Colombia-preliminary report. N. Engl. J. Med..

[9] Metsky H.C., Matranga C.B., Wohl S., Schaffner S.F., Freije C.A., Winnicki S.M., West K., Qu J., Baniecki M.L., Gladden-Young A. (2017). Zika virus evolution and spread in the Americas. Nature.

[10] Oehler E., Watrin L., Larre P., Leparc-Goffart I., Lastere S., Valour F., Baudouin L., Mallet H., Musso D., Ghawche F. (2014). Zika virus infection complicated by Guillain-Barre syndrome--case report, French Polynesia, December 2013. Eurosurveillance.

[11] Cao-Lormeau V.M., Blake A., Mons S., Lastere S., Roche C., Vanhomwegen J., Dub T., Baudouin L., Teissier A., Larre P. (2016). Guillain-Barre Syndrome outbreak associated with Zika virus infection in French Polynesia: A case-control study. Lancet..

[12] Cauchemez S., Besnard M., Bompard P., Dub T., Guillemette-Artur P., Eyrolle-Guignot D., Salje H., Van Kerkhove M.D., Abadie V., Garel C. (2016). Association between Zika virus and microcephaly in French Polynesia, 2013-15: A retrospective study. Lancet..

[13] Soares de Araujo J.S., Regis C.T., Gomes R.G., Tavares T.R., Rocha Dos Santos C., Assuncao P.M., Nobrega R.V., Pinto D.F., Bezerra B.V., Mattos S.D. (2016). Microcephaly in north-east Brazil: A retrospective study on neonates born between 2012 and 2015. Bull. World Health Organ..

[14] Liu Y., Liu J.Y., Du S.Y., Shan C., Nie K.X., Zhang R., Li X.F., Zhang R., Wang T., Qin C.F. (2017). Evolutionary enhancement of Zika virus infectivity in Aedes aegypti mosquitoes. Nature.

[15] Xia H., Luo H., Shan C., Muruato A.E., Nunes B.T.D., Medeiros D.B.A., Zou J., Xie X., Giraldo M.I., Vasconcelos P.F.C. (2018). An evolutionary NS1 mutation enhances Zika virus evasion of host interferon induction. Nat. Commun..

[16] Katzelnick L.C., Coloma J., Harris E. (2017). Dengue: Knowledge gaps, unmet needs, and research priorities. Lancet Infect. Dis..

[17] Dejnirattisai W., Supasa P., Wongwiwat W., Rouvinski A., Barba-Spaeth G., Duangchinda T., Sakuntabhai A., Cao-Lormeau V.M., Malasit P., Rey F.A. (2016). Dengue virus sero-cross-reactivity drives antibody-dependent enhancement of infection with zika virus. Nat. Immunol..

[18] Stettler K., Beltramello M., Espinosa D.A., Graham V., Cassotta A., Bianchi S., Vanzetta F., Minola A., Jaconi S., Mele F. (2016). Specificity, cross-reactivity, and function of antibodies elicited by Zika virus infection. Science.

[19] Paul L.M., Carlin E.R., Jenkins M.M., Tan A.L., Barcellona C.M., Nicholson C.O., Michael S.F., Isern S. (2016). Dengue virus antibodies enhance Zika virus infection. Clin. Transl. Immunol..

[20] Priyamvada L., Quicke K.M., Hudson W.H., Onlamoon N., Sewatanon J., Edupuganti S., Pattanapanyasat K., Chokephaibulkit K., Mulligan M.J., Wilson P.C. (2016). Human antibody responses after dengue virus infection are highly cross-reactive to Zika virus. Proc. Natl. Acad. Sci. USA.

[21] Bardina S.V., Bunduc P., Tripathi S., Duehr J., Frere J.J., Brown J.A., Nachbagauer R., Foster G.A., Krysztof D., Tortorella D. (2017). Enhancement of Zika virus pathogenesis by preexisting antiflavivirus immunity. Science.

[22] Grifoni A., Pham J., Sidney J., O’Rourke P.H., Paul S., Peters B., Martini S.R., de Silva A.D., Ricciardi M.J., Magnani D.M. (2017). Prior Dengue virus exposure shapes T cell immunity to Zika virus in humans. J. Virol..

[23] Wen J., Tang W.W., Sheets N., Ellison J., Sette A., Kim K., Shresta S. (2017). Identification of Zika virus epitopes reveals immunodominant and protective roles for dengue virus cross-reactive CD8+ T cells. Nat. Microbiol..

[24] Herrera B.B., Tsai W.Y., Chang C.A., Hamel D.J., Wang W.K., Lu Y., Mboup S., Kanki P.J. (2018). Sustained specific and cross-reactive T cell responses to Zika and Dengue virus NS3 in West Africa. J. Virol..

[25] Weiskopf D., Angelo M.A., de Azeredo E.L., Sidney J., Greenbaum J.A., Fernando A.N., Broadwater A., Kolla R.V., De Silva A.D., de Silva A.M. (2013). Comprehensive analysis of dengue virus-specific responses supports an HLA-linked protective role for CD8+ T cells. Proc. Natl. Acad. Sci. USA.

[26] Weiskopf D., Bangs D.J., Sidney J., Kolla R.V., De Silva A.D., de Silva A.M., Crotty S., Peters B., Sette A. (2015). Dengue virus infection elicits highly polarized CX3CR1+ cytotoxic CD4+ T cells associated with protective immunity. Proc. Natl. Acad. Sci. USA.

[27] Rivino L. (2016). T cell immunity to dengue virus and implications for vaccine design. Expert Rev. Vaccines.

[28] Alam A., Ali S., Ahamad S., Malik M.Z., Ishrat R. (2016). From ZikV genome to vaccine: In silico approach for the epitope-based peptide vaccine against Zika virus envelope glycoprotein. Immunology.

[29] Dar H., Zaheer T., Rehman M.T., Ali A., Javed A., Khan G.A., Babar M.M., Waheed Y. (2016). Prediction of promiscuous T-cell epitopes in the Zika virus polyprotein: An in silico approach. Asian Pac. J. Trop. Med..

[30] Usman Mirza M., Rafique S., Ali A., Munir M., Ikram N., Manan A., Salo-Ahen O.M., Idrees M. (2016). Towards peptide vaccines against Zika virus: Immunoinformatics combined with molecular dynamics simulations to predict antigenic epitopes of Zika viral proteins. Sci. Rep..

[31] Elong Ngono A., Vizcarra E.A., Tang W.W., Sheets N., Joo Y., Kim K., Gorman M.J., Diamond M.S., Shresta S. (2017). Mapping and role of the CD8+ T cell response during primary Zika virus infection in mice. Cell Host Microbe.

[32] Huang H., Li S., Zhang Y., Han X., Jia B., Liu H., Liu D., Tan S., Wang Q., Bi Y. (2017). CD8+ T cell immune response in immunocompetent mice during Zika virus infection. J. Virol..

[33] Pardy R.D., Rajah M.M., Condotta S.A., Taylor N.G., Sagan S.M., Richer M.J. (2017). Analysis of the T cell response to Zika Virus and identification of a novel CD8+ T cell epitope in immunocompetent mice. PLoS Pathog..

[34] Winkler C.W., Myers L.M., Woods T.A., Messer R.J., Carmody A.B., McNally K.L., Scott D.P., Hasenkrug K.J., Best S.M., Peterson K.E. (2017). Adaptive immune responses to Zika virus are important for controlling virus infection and preventing infection in brain and testes. J. Immunol..

[35] Aubry M., Teissier A., Huart M., Merceron S., Vanhomwegen J., Roche C., Vial A.L., Teururai S., Sicard S., Paulous S. (2017). Zika virus seroprevalence, French Polynesia, 2014–2015. Emerg. Infect. Dis..

[36] De Alwis R., Williams K.L., Schmid M.A., Lai C.Y., Patel B., Smith S.A., Crowe J.E., Wang W.K., Harris E., de Silva A.M. (2014). Dengue viruses are enhanced by distinct populations of serotype cross-reactive antibodies in human immune sera. PLoS Pathog..

[37] De Alwis R., de Silva A.M. (2014). Measuring antibody neutralization of dengue virus (DENV) using a flow cytometry-based technique. Methods Mol. Biol..

[38] Calvo E.P., Sanchez-Quete F., Duran S., Sandoval I., Castellanos J.E. (2016). Easy and inexpensive molecular detection of dengue, chikungunya and zika viruses in febrile patients. Acta Trop..

[39] Andreatta M., Karosiene E., Rasmussen M., Stryhn A., Buus S., Nielsen M. (2015). Accurate pan–specific prediction of peptide-MHC class II binding affinity with improved binding core identification. Immunogenetics.

[40] Nielsen M., Andreatta M. (2016). NetMHCpan-3.0; improved prediction of binding to MHC class I molecules integrating information from multiple receptor and peptide length datasets. Genome Med..

[41] Simmons C.P., Dong T., Chau N.V., Dung N.T., Chau T.N., Thao le T.T., Dung N.T., Hien T.T., Rowland-Jones S., Farrar J. (2005). Early T-cell responses to dengue virus epitopes in Vietnamese adults with secondary dengue virus infections. J. Virol..

[42] Duangchinda T., Dejnirattisai W., Vasanawathana S., Limpitikul W., Tangthawornchaikul N., Malasit P., Mongkolsapaya J., Screaton G. (2010). Immunodominant T-cell responses to dengue virus NS3 are associated with DHF. Proc. Natl. Acad. Sci. USA.

[43] Rivino L., Kumaran E.A., Jovanovic V., Nadua K., Teo E.W., Pang S.W., Teo G.H., Gan V.C., Lye D.C., Leo Y.S. (2013). Differential targeting of viral components by CD4+ versus CD8+ T lymphocytes in dengue virus infection. J. Virol..

[44] Weiskopf D., Angelo M.A., Bangs D.J., Sidney J., Paul S., Peters B., de Silva A.D., Lindow J.C., Diehl S.A., Whitehead S. (2015). The Human CD8+ T Cell Responses Induced by a Live Attenuated Tetravalent Dengue Vaccine Are Directed against Highly Conserved Epitopes. J. Virol..

[45] Dikhit M.R., Ansari M.Y., Vijaymahantesh, Kalyani, Mansuri R., Sahoo B.R., Dehury B., Amit A., Topno R.K., Sahoo G.C. (2016). Computational prediction and analysis of potential antigenic CTL epitopes in Zika virus: A first step towards vaccine development. Infect. Genet. Evol..

[46] Imrie A., Meeks J., Gurary A., Sukhbataar M., Kitsutani P., Effler P., Zhao Z. (2007). Differential functional avidity of dengue virus-specific T-cell clones for variant peptides representing heterologous and previously encountered serotypes. J. Virol..

[47] Tai W., He L., Wang Y., Sun S., Zhao G., Luo C., Li P., Zhao H., Fremont D.H., Li F. (2018). Critical neutralizing fragment of Zika virus EDIII elicits cross-neutralization and protection against divergent Zika viruses. Emerg. Microbes Infect..

[48] Collins M.H., McGowan E., Jadi R., Young E., Lopez C.A., Baric R.S., Lazear H.M., de Silva A.M. (2017). Lack of durable cross-neutralizing antibodies against Zika virus from Dengue virus infection. Emerg. Infect. Dis..

[49] Katzelnick L.C., Gresh L., Halloran M.E., Mercado J.C., Kuan G., Gordon A., Balmaseda A., Harris E. (2017). Antibody-dependent enhancement of severe dengue disease in humans. Science..

[50] Priyamvada L., Hudson W., Ahmed R., Wrammert J. (2017). Humoral cross-reactivity between Zika and dengue viruses: Implications for protection and pathology. Emerg. Microbes Infect..

[51] Zhang S.C., Martin E., Shimada M., Godfrey S.B., Fricke J., Locastro S., Lai N.Y., Liebesny P., Carlson J.M., Brumme C.J. (2012). Aminopeptidase substrate preference affects HIV epitope presentation and predicts immune escape patterns in HIV-infected individuals. J. Immunol..

[52] Ricciardi M.J., Magnani D.M., Grifoni A., Kwon Y.C., Gutman M.J., Grubaugh N.D., Gangavarapu K., Sharkey M., Silveira C.G.T. (2017). Ontogeny of the B- and T-cell response in a primary Zika virus infection of a dengue-naive individual during the 2016 outbreak in Miami, FL. PLoS Negl. Trop. Dis..

[53] Swanstrom J.A., Plante J.A., Plante K.S., Young E.F., McGowan E., Gallichotte E.N., Widman D.G., Heise M.T., de Silva A.M., Baric R.S. (2016). Dengue virus envelope dimer epitope monoclonal antibodies isolated from dengue patients are protective against Zika virus. MBio.

[54] Morens D.M., Halstead S.B. (1990). Measurement of antibody-dependent infection enhancement of four dengue virus serotypes by monoclonal and polyclonal antibodies. J. Gen. Virol..

[55] Guy B., Chanthavanich P., Gimenez S., Sirivichayakul C., Sabchareon A., Begue S., Yoksan S., Luxemburger C., Lang J. (2004). Evaluation by flow cytometry of antibody-dependent enhancement (ADE) of dengue infection by sera from Thai children immunized with a live-attenuated tetravalent dengue vaccine. Vaccine.

[56] Barba-Spaeth G., Dejnirattisai W., Rouvinski A., Vaney M.C., Medits I., Sharma A., Simon-Loriere E., Sakuntabhai A., Cao-Lormeau V.M., Haouz A. (2016). Structural basis of potent Zika-dengue virus antibody cross-neutralization. Nature.

[57] Robbiani D.F., Bozzacco L., Keeffe J.R., Khouri R., Olsen P.C., Gazumyan A., Schaefer-Babajew D., Avila-Rios S., Nogueira L., Patel R. (2017). Recurrent potent human neutralizing antibodies to Zika virus in Brazil and Mexico. Cell.

[58] Rogers T.F., Goodwin E.C., Briney B., Sok D., Beutler N., Strubel A., Nedellec R., Le K., Brown M.E., Burton D.R. (2017). Zika virus activates de novo and cross-reactive memory B cell responses in dengue-experienced donors. Sci. Immunol..

[59] McCracken M.K., Gromowski G.D., Friberg H.L., Lin X., Abbink P., De La Barrera R., Eckles K.H., Garver L.S., Boyd M., Jetton D. (2017). Impact of prior flavivirus immunity on Zika virus infection in rhesus macaques. PLoS Pathog..

[60] Pantoja P., Perez-Guzman E.X., Rodriguez I.V., White L.J., Gonzalez O., Serrano C., Giavedoni L., Hodara V., Cruz L., Arana T. (2017). Zika virus pathogenesis in rhesus macaques is unaffected by pre-existing immunity to dengue virus. Nat. Commun..

[61] Dan J.M., Lindestam Arlehamn C.S., Weiskopf D., da Silva Antunes R., Havenar-Daughton C., Reiss S.M., Brigger M., Bothwell M., Sette A., Crotty S. (2016). A Cytokine-Independent Approach To Identify Antigen-Specific Human Germinal Center T Follicular Helper Cells and Rare Antigen-Specific CD4+ T Cells in Blood. J. Immunol..

