# Supplementary material for: Improved Immune Responses Against Zika Virus After Sequential Dengue and Zika Virus Infection in Humans
Source: Viruses. 2018 Sep 7;10(9):480. doi: 10.3390/v10090480 (PMC6164826; doi:10.3390/v10090480)
Supplement: Supplementary file 1 [file viruses-10-00480-s001.zip › Fig_S1.pdf]

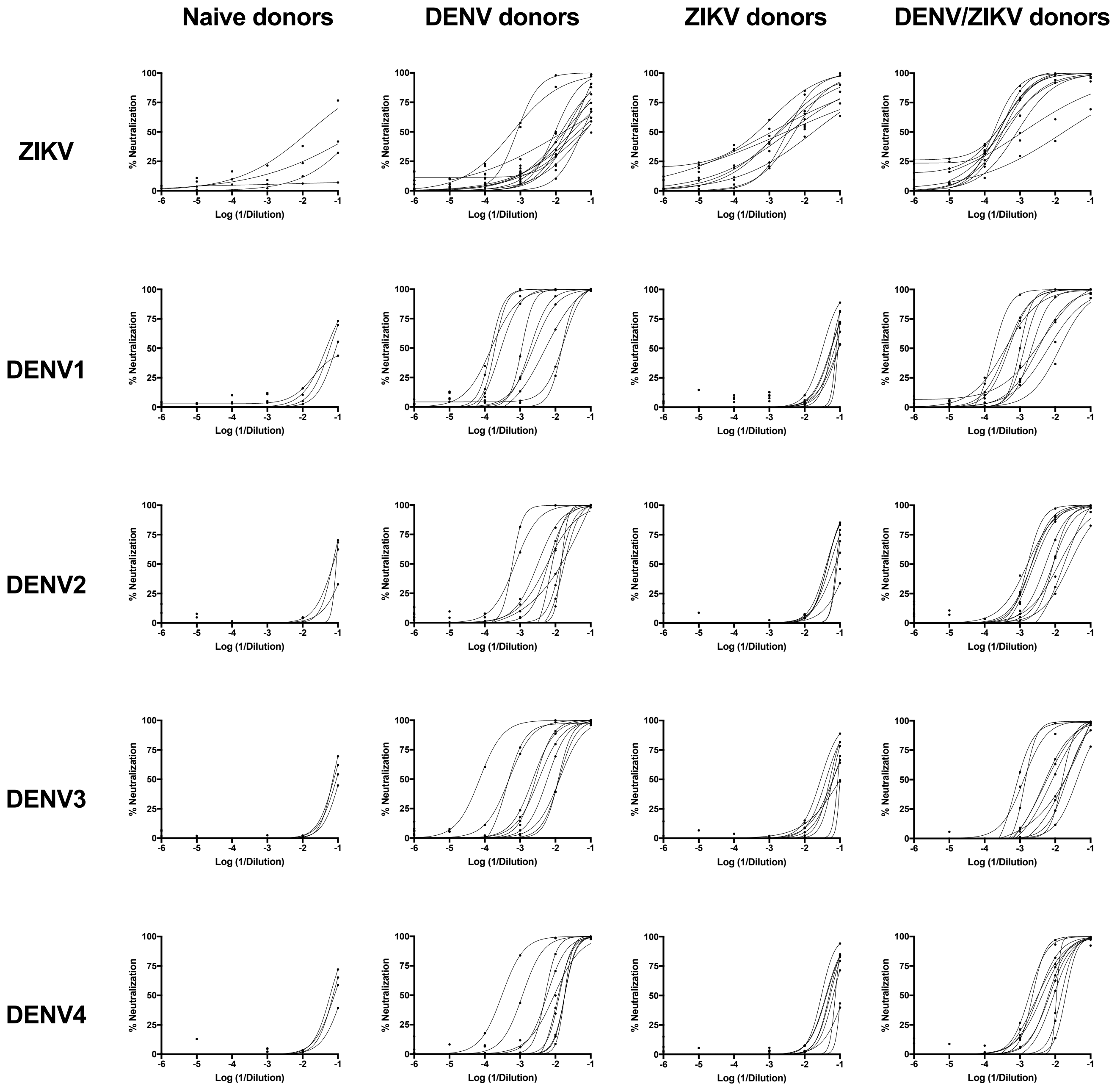

Naive donors
DENV donors
ZIKV donors
DENV/ZIKV donors
ZIKV
DENV1
DENV2
DENV3
DENV4
% Neutralization
Log (1/Dilution)

Fig. S1: Comparison of the neutralization activity against ZIKV and DENV1-4 infection between plasma samples from all donors in the study cohort. Neutralization activity against ZIKV and DENV infection using plasma samples from Naïve donors (left panels), DENV-immune ZIKV-naïve (group A) donors (DENV donors, middle left panels), ZIKV-immune DENV-naïve (group B) donors (ZIKV donors, middle right panels) and DENV/ZIKV-immune (groups C, D and E) donors (DENV/ZIKV donors, right panels).
