# Supplementary material for: Improved Immune Responses Against Zika Virus After Sequential Dengue and Zika Virus Infection in Humans
Source: Viruses. 2018 Sep 7;10(9):480. doi: 10.3390/v10090480 (PMC6164826; doi:10.3390/v10090480)
Supplement: Supplementary file 1 [file viruses-10-00480-s001.zip › TABLE_S1.pdf]

Table S1. Characteristics of antigenic peptides from ZIKV identified in this study

Peptide[a]	Sequence[b]	Donors[c]	HLA[d]	SFC/million PBMC[e] 15-mer	9-mer	Predicted epitope	Predicted HLA	Score (rank)[f]
C_{13-27}	IVNM**LKRGVARVS**PF	28 (C)	A**02**,29; B40,44; C03,16; DRB104,07	170	<20	MLKRGVARV	A0217	1.9
		60 (B)	A**02**,69; B55,58; C01,07; DRB111,13	50	60		A0205	1.3
C_{85-99}	KKDL**AAMLRIINA**RK	26 (D)	A**02**,24; B07,48; C01,07; DRB114,15	100	<20	AAMLRIINA	A0201	4.5
		60 (B)	A**02**,69; B55,58; C01,07; DRB111,13	<20	75	KDLAAMLRI	B5501	1.4
	K**KDLAAMLRI**INARK	28 (C)	A**02**,29; B40,44; C03,16; DRB104,07	230	65		B4002	2.0
$E_{455-469}$	GMSWFSQILIGTLLM	1 (B)	A**02**,24; B35,**51**; C01,01; DRB104,12	120	NT	GMSWFSQILI	A0201	0.9
		77 (B)	A**02,02**; B40,**51**; C04,07; DRB113,14	35	NT	MSWFSQILI	B5101	0.12
$NS1_{63-77}$	MEN**IMWRSVEGE**LNA	21 (B)	A31,03; B35,18; C01,17; DRB1**04**,03	65	50	MENIMWRSVEGELNA	DRB10405	50
	MENIM**WRSVEGELN**A	28 (C)	A**02**,29; B40,44; C03,16; DRB1**04**,07	245	58	IMWRSVEGEL	A0217	0.5
		56 (D)	A**02,02**; B15,18; C05,07; DRB103,11	70	148		A0201	1.2
$NS1_{83-97}$	G**VQLTVVVGS**VKNPM	26 (D)	A**02**,24; B07,48; C01,07; DRB114,15	145	23	VQLTVVVGSV	A0201	1.7
		28 (C)	A**02**,29; B40,44; C03,16; DRB104,07	165	35		A0217	3
$NS1_{163-177}$	FHTS**VWLKVREDY**SL	28 (C)	A02,29; B40,44; C03,16; DRB1**04**,07	230	75	HTSVWLKVREDY	A0101	0.4
	FHTSV**WLKVREDYS**L	55 (C)	A03,11; B35,51; C04,15; DRB101,07	110	125	HTSVWLKVR	A3101	0.4
		46 (B)	A32,68; B39,50; C06,07; DRB1**04**,07	50	32		A6801	0.6
		20 (E)	A01,31; B07,39; C07,07; DRB1**04**,15	310	105	VWLKVREDY	A2902	1.3
						FHTSVWLKV	B3905	0.4
							B3901	0.4
$NS1_{275-289}$	IRFEE**CPGTKVHVE**E	33 (C)	A24,24; B15,**35**; C01,03; DRB104,04	215	55	CPGTKVHVE	B3501	8.5
		55 (C)	A03,11; B**35**,51; C04,15; DRB101,07	130	115		B3531	6.5

Continued

Table S1. Characteristics of antigenic peptides from ZIKV identified in this study – cont'd

Peptide[a]	Sequence[b]	Donors[c]	HLA[d]	SFC/million PBMC[e] 15-mer	SFC/million PBMC[e] 9-mer	Predicted epitope	Predicted HLA	Score (rank)[f]
NS2B$_{117-131}$	AAGAWYVYVKTGKRS	55 (C)	A03,11; B35,51; C04,15; DRB101,07	445	NT	AAGAWYVYVK	A0301	0.6
							A1101	0.12
						YVYVKTGKR	A0301	1.8
NS3$_{219-233}$	TVILAPTRVVAAEME	53 (D)	A23,31; B**40**,44; C01,04; DRB107,**08**	100	NT	TVILAPTRVVAAEME	DRB10802	1.5
		66 (C)	A02,03; B39,**40**; C03,07; DRB1**08**,15	65	NT	ILAPTRVVAA	A0201	1.6
NS3$_{271-285}$	LQPIRVPNYNLYIMD	42 (B)	A26,26; B35,38; C05,06; DRB104,11	165	NT	VPNYNLYIM	B3501	0.06
NS3$_{311-325}$	AAI**FMTATPPGT**RDA	28 (C)	A02,29; B40,44; C03,16; DRB1**04**,07	255	85	AAIFMTATPPGTRDA	DRB10401	4
						FMTATPPGT	A0217	5.5
	AA**IFMTATPPG**TRDA	33 (C)	A24,24; B15,35; C01,03; DRB1**04**,04	215	30	IFMTATPPG	A2402	5
NS4A$_{86-100}$	VTLGASAWLMWLSEI	55 (C)	A03,11; B35,51; C04,15; DRB101,07	178	NT	SAWLMWLSEI	B5101	0.9
		60 (B)	A02,69; B55,58; C01,07; DRB111,13	125	NT	VTLGASAWL	A6901	1.3
						LGASAWLMW	B5801	0.07
NS4B$_{112-126}$	**AIILLVAHY**MYLIPG	28 (C)	A**02**,29; B40,44; C03,16; DRB104,07	60	58	AIILLVAHY	A2902	0.6
		37 (B)	A**02**,11; B35,50; C04,06; DRB101,13	75	30		A1101	3.5
	AII**LLVAHYMYL**IPG	60 (B)	A**02**,69; B55,58; C01,07; DRB111,13	100	68	LLVAHYMYL	A0205	0.3
	AIIL**LVAHYMYLI**PG	60 (B)	A**02**,69; B55,58; C01,07; DRB111,13	100	35	LVAHYMYLI	A6901	0.15
							A0205	0.2
NS5$_{13-27}$	KARLNQMSALEFYSY	55 (C)	A03,11; B**35**,51; C04,15; DRB101,07	260	NT	MSALEFYSY	B3501	0.15
		69 (E)	A01,24; B**35,35**; C01,04; DRB104,13	145	NT		A0101	0.09

Continued

Table S1. Characteristics of antigenic peptides from ZIKV identified in this study – cont'd

Peptide[a]	Sequence[b]	Donors[c]	HLA[d]	SFC/million PBMC[e] 15-mer	9-mer	Predicted epitope	Predicted HLA	Score (rank)[f]
NS5$_{293-307}$	WFFDEN**HPYRTWAYH**	55 (C)	A03,11; B**35**,51; C04,15; DRB101,07	1580	308	HPYRTWAYH	B3501	0.4
	W**FFDENHPYR**TWAYH	69 (E)	A01,24; B**35,35**; C01,04; DRB104,13	40	218	FFDENHPY	A0101	1.6
NS5$_{297-311}$	E**NHPYRTWAY**HGSYE	55 (C)	A03,11; B**35**,51; C04,15; DRB101,07	1280	358	NHPYRTWAY	B3501	3
	ENHP**YRTWAYHGS**YE	69 (E)	A01,24; B**35,35**; C01,04; DRB104,13	75	188	YRTWAYHGSY	B3501	1.7
							A0101	0.3
	ENHPY**RTWAYHGSY**E	69 (E)	A01,24; B**35,35**; C01,04; DRB104,13	75	205	RTWAYHGSY	A0101	0.5
NS5$_{345-359}$	TDT**TPYGQQRVF**KEK	33 (C)	A24,24; B15,**35**; C01,03; DRB104,04	1315	395	TPYGQQRVF	B3531	0.7
		55 (C)	A03,11; B**35**,51; C04,15; DRB101,07	2095	523		B3501	0.3
		69 (E)	A01,24; B**35**,35; C01,04; DRB104,13	785	763			
NS5$_{425-439}$	**EAVNDPRFW**ALVDKE	28 (C)	A02,29; B40,44; C03,16;DRB104,07	150	100	AVNDPRFWALVDK	A0301	1.1
		55 (C)	A03,11; B35,51; C04,15; DRB101,07	120	125		A1101	0.6
		56 (D)	A02,02; B15,18; C05,07; DRB103,11	90	240			
NS5$_{461-475}$	KKQGEFGKAKGSRAI	28 (C)	A02,29; B**40**,44; C03,16;DRB104,**07**	300	NT	KKQGEFGKAKGSRAI	DRB10701	32
		53 (D)	A23,31; B**40**,44; C01,04; DRB1**07**,08	105	NT	GEFGKAKGSRAI	B4002	0.7
NS5$_{473-487}$	RAIWYMWLGARFLEF	28 (C)	A02,29; B40,44; C03,16; DRB104,**07**	210	NT	YMWLGARFL	A0217	0.03
		55 (C)	A03,11; B35,51; C04,15; DRB101,**07**	295	NT	AIWYMWLGAR	A0301	1.3
						RAIWYMWLGARFLEF	DRB10701	16

Continued

Table S1. Characteristics of antigenic peptides from ZIKV identified in this study – cont'd

Peptide[a]	Sequence[b]	Donors[c]	HLA[d]	SFC/million PBMC[e]		Predicted epitope	Predicted HLA	Score (rank)[f]
				15-mer	9-mer			
NS5$_{546-560}$	RFDLENEALITNQME	28 (C)	A02,29; B**<u>40</u>**,44; C03,16; DRB104,07	245	NT	NEALITNQM	B4002	0.8
		53 (D)	A23,31; B**<u>40</u>**,44; C01,04; DRB107,08	190	NT		B4403	0.6
		66 (C)	A02,03; B39,**<u>40</u>**; C03,07; DRB108,15	80	NT		B3901	1.8
NS5$_{565-579}$	LALAIIKYTYQNKVV	28 (C)	A02,29; B**<u>40</u>**,44; C03,16; DRB104,07	240	NT	LALAIIKYTY	A2902	0.5
		53 (D)	A23,31; B**<u>40</u>**,44; C01,04; DRB107,08	120	NT	ALAIIKYTY	A2902	0.25
		56 (D)	A02,02; B15,18; C05,07; DRB103,11	150	NT	LALAIIKYTY	B1517	1.2
NS5$_{605-619}$	QVVT**<u>YALNTFTNL</u>**VV	33 (C)	A**<u>24,24</u>**; B15,35; C01,03; DRB104,04	240	NT	TYALNTFTNL	A24:02	0.09
		59 (B)	A03,**<u>24</u>**; B35,40; C01,03; DRB104,04	50	42	YALNTFTNL	B35:43	0.4
							B35:31	0.25

[a] The position of peptides were determined according to NCBI Reference Sequence YP_002790881.1; [b] The underlined and in bold sequence correspond to the 9-mer peptide tested; [c] The letters in brackets refer to the group of donors indicated in Table 1; [d] The common alleles between donors are underlined and in bold; [e] Cumulative SFC/million PBMC; NT, not tested; [f] Calculated using NetMHCpan 3.0 Server: strong binders <0.5, weak binders <2.
